# An Interdisciplinary Intervention Based on Prescription of Physical Activity, Diet, and Positive Mental Health to Promote Healthy Lifestyle in Patients with Obesity: A Randomized Control Trial

**DOI:** 10.3390/nu16162776

**Published:** 2024-08-20

**Authors:** Marta Prats-Arimon, Montserrat Puig-Llobet, Oriol Barceló-Peiró, Ivet Ribot-Domènech, Cristina Vilalta-Sererols, Bárbara Fontecha-Valero, Mati Heras-Ojeda, Zaida Agüera, Teresa Lluch-Canut, Antonio Moreno-Poyato, Mª Carmen Moreno-Arroyo

**Affiliations:** 1 Department of Fundamental and Medical-Surgical Nursing, Faculty of Nursing, University of Barcelona, 08036 Barcelona, Spain; mprats@hcerdanya.eu (M.P.-A.); carmenmoreno@ub.edu (M.C.M.-A.); 2 Sports Medicine Department, Hospital Transfronterer de Cerdanya (AECT), Puigcerdà, 17520 Girona, Spain; obarcelo@hcerdanya.eu (O.B.-P.); iribot@hcerdanya.eu (I.R.-D.); 3 Department of Public Health, Mental Health and Maternal-Child Nursing, Faculty of Nursing, University of Barcelona, 08036 Barcelona, Spain; zaguera@ub.edu (Z.A.); tlluch@ub.edu (T.L.-C.); amorenopoyato@ub.edu (A.M.-P.); 4 Primary Care Centre, Fundació Hospital de Puigcerdà, Puigcerdà, 17520 Girona, Spain; cvilalta@fhp.cat (C.V.-S.); bfontecha@fhp.cat (B.F.-V.); 5 Sports Council of Cerdanya, Girona Provincial Council, 17004 Girona, Spain; mheras.esport@cerdanya.org

**Keywords:** prescription on physical activity, obesity, diet, Positive Mental Health, nutrition, improved lifestyle

## Abstract

This study used a multimodal approach to address the issue of obesity among individuals with a Body Mass Index (BMI) between 30 and 40 residing in a rural region of north-east Spain. A pretest–posttest model was employed in a clinical trial design, comparing an intervention group with a control group. The intervention, which lasted for a period of nine months, was based on three main strategies: the prescription of physical activity, the promotion of healthy nutritional habits, and the management of emotional wellbeing through Positive Mental Health (PMH). A variety of assessment tools were employed, including the CLASS-AF scale and a stress test for physical activity levels; advanced anthropometry and blood analysis for metabolic and body composition variables; a Mediterranean diet adherence questionnaire for nutritional habits; and a PMH multifactorial questionnaire for the assessment of emotional management. The results revealed significant improvements in the level of physical activity and adherence to the Mediterranean diet in favor of the intervention group, where 89.4% (n = 17) of the participants went from being not very active/sedentary to being active. Also, adherence to the Mediterranean diet improved with a mean increase of 2.2 points on the scale [mean: 10.5 (CI 95%: 9.90, 11.09)]. In addition, significant reductions in body fat [mean: −2.50 kg (CI 95%: −3.56, −1.44)] and free fat mass [mean: −3.38% (IC 95%: −4.34, −2.41), along with decreased cholesterol levels (196 vs. 182 mg/dL), were observed, suggesting a decrease in cardiovascular and metabolic risk. In conclusion, this multimodal intervention was effective at improving the lifestyle of people with obesity and reducing their cardiovascular and metabolic risk. The combination of interventions focused on physical activity, diet, Positive Mental Health, and metabolic changes and were perceived as a comprehensive and complementary strategy in obesity care. These findings highlight the importance of approaching this condition from multiple perspectives to ensure optimal health outcomes.

## 1. Introduction

The World Health Organization (WHO) has identified obesity and sedentary lifestyles or physical inactivity as significant public health concerns [1,2,3]. There is a correlation between obesity and non-communicable diseases (NCDs), including ischemic heart disease [4], stroke [5,6], cancer [7,8], diabetes [9], and others [10,11,12]. Obesity represents one of the principal causes of mortality in the 21st century [13,14,15,16]. Obesity currently affects 30% of women and 35% of men, and 49.7% of the global population is sedentary [17]. A sedentary person is defined as someone who engages in prolonged periods of sitting, exceeding five hours per day andexhibits a metabolic energy expenditure that does not substantially increase metabolic energy expenditure above the basal resting level [18]. Sedentary behaviour includes activities such as sleeping, sitting, lying down, and watching television and other forms of screen-based entertainment [19]. The combination of sedentary behavior and physical inactivity (which was described as performing insufficient amounts of physical activity, that is, not meeting specified physical activity guidelines by the WHO [20]) is associated with an elevated risk of non-communicable diseases [21]. The most recent survey conducted by the National Health System in Spain (2017) revealed that 54.5% of adults were affected by obesity or overweight, with rates reaching 27.8% among children and adolescents aged between 2 and 17 years old. Furthermore, 40.3% of Spanish women and 32.3% of Spanish men are sedentary, particularly within the 35–44 and 65+ age groups in the adult population. The prevalence of physical activity is higher among men, with 55% of men engaging in physical exercise for more than one day per week. Regarding dietary habits, 33% of the Spanish population do not consume fruit on a daily basis, and 64% do not eat vegetables daily. Women tend to exhibit superior nutritional habits compared to men [21].

The term ‘physical activity’ is defined as any muscular movement that requires energy expenditure [22], measured by the metabolic equivalent of task (MET), which is equivalent to the amount of oxygen consumed while sitting at rest and is equal to 3.5 mL O_2_ per kg body weight x min. This includes regular activities of daily life that involve body movement such as walking or climbing stairs [23]. Conversely, physical exercise can be defined as a planned effort to improve people’s fitness, health, and quality of life [24]. A regular exercise program includes cardiorespiratory endurance, flexibility, and neuromotor exercise training. The American College of Sports Medicine and the WHO recommend adults to perform moderate intensity physical exercise 30 min/day for five days a week, or vigorous intensity physical exercise for 20 min/day, three days a week [25].

It has been scientifically proven that physical activity with a certain frequency and intensity leads to health benefits [26,27] and improves people’s physical [24,27], psychological, biological, and social well-being, favoring the reduction of cardiovascular risk factors for NCDs [13,28] such as such as arterial hypertension [29], and metabolic risk factors such as diabetes or obesity [30], increasing Positive Mental Health [31,32] and strengthening the musculoskeletal system [33].

At present, the introduction of specialized technology and new technologies has led to changes in lifestyle, favoring unstructured meals and eating habits, especially in vulnerable population groups, such as people with low income and low educational level, people with emotional or mental disorders, and adolescents [34,35]. Furthermore, men are at a higher risk of developing obesity than women, largely due to their less healthy lifestyles [36]. This has led to the consumption of fast food, pastries, and sugars, as well as a corresponding increase in sedentary lifestyles associated with screen time, which in turn has led to an increase in stress and anxiety levels [37]. These determinants have a direct impact on public health [34,38]. In this context, since 2010, the WHO has been developing policies at all levels (state, community, regional, and local) with the aim of preventing obesity and sedentary lifestyles [17,39,40]. These policies address not only the issue of recommended physical activity but also mental health and healthy eating habits based on the Mediterranean diet, which is characterized by the daily consumption of fruit and vegetables, legumes, nuts, cereals, fish, and olive oil, as well as a reduction in the consumption of red meat [41,42]. The 2030 action plan outlines a comprehensive intervention strategy at all levels to address the comorbidity of non-communicable disease (NCD) [43]. In a similar vein, a number of studies have employed an interdisciplinary approach to the treatment of obesity, yielding more favorable outcomes than those achieved through single-modality interventions [44,45]. It is therefore estimated that individuals with obesity are more prone to developing emotional disorders, including anxiety, stress, and depression [46,47]. Furthermore, the evidence to date shows that physical exercise has a beneficial impact on mental health outcomes [31,32,48]. The implementation of self-care strategies and the advancement of Positive Mental Health outcomes have been demonstrated to enhance the adherence of individuals with obesity to a healthy lifestyle [45,49]. The incorporation of Positive Mental Health elements into obesity-related programs facilitates an enhanced perception of health status, thereby favoring resilience to potential adversities associated with the disease [50]. The principal aim was to assess the effectiveness of a multidisciplinary health program called “Cerdanya en Forma” (Cerdanya in Fit), addressed at people with obesity. The specific aims were fourfold: (a) to examine whether the program improves the physical activity levels and, therefore, physical condition; (b) to analyze whether this intervention increases the levels of adherence to the Mediterranean diet; (c) to explore whether the intervention helps to enhance the Positive Mental Health in this target population; and (d) to describe the metabolic changes that take place as a clinical outcome of this intervention.

## 2. Materials and Methods

### 2.1. Design

This study was designed in two phases: Phase I: a descriptive, cross-sectional observational study; and Phase II: a clinical trial in which the participants of phase I were randomized into two groups: a control group and an intervention group following a pre-post-test model. Randomization was performed using a database that assigned phase I participants a number, either 1 or 2, alternately stratified by gender. In this phase, the intervention “Cerdanya en Forma” was carried out, aimed at people with mild to moderate obesity to promote a healthy lifestyle and reduce comorbidity. The duration of the study was nine months, and it was carried out in the primary care area, the Sports Medicine and Physical Activity Unit, and the nutrition unit of the Hospital of Cerdanya, all in Cerdanya, a rural community located in the north-east of Spain.

The study was conducted according to the Declaration of Helsinki statement. Ethical approval was obtained from the Arnau de Vilanova Hospital Committee-Cataluña-Spain (Protocol 2038/2019). All participants voluntarily gave informed, written consent. The study was registered at https://register.clinicaltrials.gov/prs/app/action/LoginUser?ts=1&cx=-jg9qo4 on 7 June 2022 and was retrospectively registered. The study was conducted and is reported in accordance with the CONSORT 2010 guidelines.

### 2.2. Participants

The study population were people with mild-moderate obesity, i.e., with a Body Mass Index (BMI) of 30–40, assigned to the Primary Care Centre of Puigcerdà with an allocation of 512 people. In phase I, the sample selection was based on consecutive cases for a total of 81 participants. In phase II, participants from phase I were randomized with a 1:1 ratio into two groups, either to receive the “Cerdanya en Forma” intervention (intervention group) or to follow the advice and recommendations of the Primary Care Centre nurse (control group). The number of participants enrolled in phase 2 was n = 60, i.e., 30 people in the intervention group and 30 in the control group. The sample calculation was made accepting an alpha risk of 0.05 and a power of 80, to detect a 25% difference between the study groups with an estimated 10% loss.

Inclusion criteria: Phase I: Adults (18–75 years of age) assigned to the Primary Care Centre where the study was conducted with people diagnosed with mild-moderate obesity (BMI 30–40). Phase II: Adults (18–75 years of age) assigned to the Primary Care Centre where the study was carried out with mild-moderate obesity (BMI 30–40), sedentary or poorly active, who agreed to participate in the program (attending the directed physical sessions, psycho-emotional sessions, and medical and nutritional check-ups) after receiving a written explanation and information about the program. Exclusion criteria: Phase I: individuals with profound hearing impairments and/or those with severe visual disabilities, such as over 50% of sight loss and requiring assistive devices for mobility. Individuals with mental or cognitive disorders in addition to obesity. Phase II: individuals with morbid obesity (BMI > 40), individuals with overweight (BMI < 30), individuals who did not comply with the therapeutic commitment and/or informed consent, and individuals presenting any medical condition that precludes the practice of physical activity and thus renders the program inapplicable.

### 2.3. Intervention

The “Cerdanya en Forma” intervention was developed over a nine-month period and consisted of three strategies:

Physical activity strategy: coordinated by a Sports Medicine and Physical Activity Unit and a professional with a degree in sports science and physical activity. It consisted of an individualized prescription of physical activity based on three items: mobility (measured by steps/day), physical exercise (measured by intensity based on the heart rate measured in the stress test), and sedentary lifestyle (sitting for less than 1:30 h at a time, taking an active break (getting up, moving around, etc. for 10 min) to break the sedentary time) where achievable objectives were agreed upon for the participant. Mobility was recorded using the Google FIT APP, a step counter software for mobile phones, which was developed by the WHO to record physical activity, or with a Decathlon W500M heart rate and step counter wristband. Also, the autonomous physical exercise guidelines were based on the WHO physical activity recommendations for adult people and on the results of the stress test performed in the initial program assessment. A notebook was used for participants to record their perception of effort using the Borg scale, frequency in days, duration in minutes, type of activity, and average Heart Rate. The participants of the intervention group received a follow-up visit every two months with the nurse of the Sports Medicine Unit, where the mobility, physical exercise, and walking goals agreed upon in the previous visits were evaluated.

Furthermore, the program participants engaged in a weekly physical activity session with a duration of 60 min. The session was led by the sports technician and alternated aerobic exercise, such as Nordic walking (walking with poles), with strength, flexibility and muscle toning exercises for the upper limbs, trunk, and lower limbs, using resistance bands and body weight. The session alternated between Nordic walking and muscle toning, as well as strength and flexibility exercises, which took place at a fitness center.

Nutritional strategy: a nutritionist performed an initial nutritional assessment of each patient, including a food frequency intake questionnaire and a 24-h recall log to determine specific goals for each patient. Food intake planning was based on the Mediterranean dietary guidelines of three to four servings of fruit and vegetables per day; the consumption of legumes, nuts, cereals, fish, and olive oil; and the reduced consumption of red meat. The quantities and portions were measured in grams. A follow-up was performed every two months where subjects were weighed, and the objectives were assessed. In addition, a bioimpedance test was performed on the third and sixth month of the program. 

Positive Mental Health (PMH) strategy: four 90-min psycho-emotional sessions were held throughout the intervention. These sessions were based on dynamics and activities to enhance personal satisfaction, autonomy, conflict resolution, proactive attitude, self-control, and interpersonal relationship skills. Relaxation, conversation, and body awareness techniques were used.

### 2.4. Variables and Instruments

An ad hoc form was used to collect sociodemographic variables (gender, age, marital status, number of children, educational level, profession, and origin) and health variables (weight, height, BMI, time of diagnosis of obesity, diabetes, hypertension, dyslipidemia, ischemic heart disease, medication, tobacco use, and beta-blocker use).

Specific health variables:

A blood test was performed by venipuncture to obtain a lipid profile (High-Density Lipoprotein (HDL), Low-Density Lipoprotein (LDL), total cholesterol, and triglycerides) and glucose pre-post test in the intervention group.

Cardiovascular risk was calculated with the waist–hip index, and metabolic risk was calculated with the waist–height index.

Nutrition and body composition variables:

Food frequency intake questionnaire (ad hoc): Questionnaire based on different food groups assessing the daily and weekly frequency of each group: dairy products were measured in 50 g or 200 cc portions, protein (eggs, meat, and fish) in 150 g portions, vegetables in 200 g portions, fruit in one or two pieces per portion, and nuts in 30 g portions. Cereals and pulses were measured in 40 g portions for bread or 150 g portions for pulses. Fats such as oils were measured in tablespoons and pastries in (50 g/serving). Sugary drinks and juices were measured in (200 mL/serving) and alcoholic drinks in (100 mL/serving).

Hour dietary recall: Participants provided a detailed 24-h recall of all foods and beverages consumed in the previous day. This recall provided a snapshot of their dietary intake.

The Mediterranean diet adherence questionnaire, validated by Martin-Moreno et al. (1993), was employed. The questionnaire comprises 14 items pertaining to the consumption of fruit, vegetables, meat, sugars, nuts, and olive oil, with measurements expressed in grams, servings per day, or number of tablespoons. A score of 0 or 1 is assigned if the minimum recommended servings are met. A total score of greater than nine is indicative of poor adherence to the Mediterranean diet [51].

Bioimpedance: The TANITA DC 240 MA with software suitebiologica pro scale was used to measure weight (kg), % water, % fat, and % free fat.

Advanced anthropometry: The advanced anthropometry was performed by a person trained in the International Society for the Advancement of Kinanthropometry (ISAK) Method. The anthropometric values measured were: weight (kg), height (cm), and BMI using an analogue scale; three skinfolds (triceps, quadriceps, and calf) using a skinfold caliper (Harper model); three perimeters (waist 1, waist 2, and hip) measured using an anthropometric metal tape; and three diameters (wrist, elbow, and femur) measured using a pachymeter. The specific formulas for people with obesity were used to calculate the percentage and kg of fat (Weltmann), muscle mass (Lee), and bone mass (Rocha) [52]. 

Physical activity variables:

The level of physical activity was assessed with the CLASS AF scale. This scale assesses physical activity at work or at home and physical activity in leisure time. Physical activity at work or at home is classified as inactive, sitting most of the day (METs equivalence > 1.2); light, standing most of the day (>2 METs); moderate, frequent walking (>3 METs); and intense, activity requiring significant physical exertion (>5 METs). Leisure-time physical activity is classified according to the intensity of the exercise as follows: light (>3 METs) activities such as yoga, Pilates, etc.; moderate (>5 METs) activities such as swimming, cycling, and gymnastics; or intense (>7 METs) activities such as football, basketball, and hockey. Based on the frequency (days x week) and minutes x day of physical activity performed, the formula (2 × L + Llx freq2) is applied. From a quantitative estimation, a qualitative recoding can be made, where 0–1 is considered sedentary, 2–3 is minimally active, 4–5 is slightly active, 6–11 is moderately active, and >12 is very active [25]. 

Physical fitness was estimated by performing a gas mask treadmill exercise test using the cardio Bruce protocol, consisting of 3-min stages of varying speed and incline [53]. Heart rate, expiratory volume, volume of oxygen consumed, and volume of CO_2_ were continuously monitored. Blood pressure was measured at the beginning and end of the test. The test was considered maximal when the person reached an HR > 90% of the maximal HR or when the patient reached exhaustion. The aforementioned parameters collected during the test determined the aerobic and anaerobic capacity of the participants, and the blood pressure curve (mmHg) and maximum heart rate (beats per minute) during exercise were estimated. Maximal oxygen uptake (VO2Max) in ml/kg.min and range of perceived exertion (RPE) [1,2,3,4,5,6,7,8,9,10] during exercise were also collected.

Positive Mental Health variables:

The Positive Mental Health Questionnaire (PMHQ) is designed to assess mental health from a positive perspective. This multifactorial model is based on six factors (self-esteem, self-control, proactive attitude, conflict resolution, personal satisfaction, and interpersonal relationship skills). This scale consists of 39 items, formulated in positive and negative, where 1 is always or almost always; 2, quite often; 3, sometimes; and 4, always or almost always. The score ranges from 39 points to 156 points. A low level of Positive Mental Health is considered when the score is 39–79 points, medium when it is 79–117 points, and high when it is 118–156 points. A higher score is indicative of greater Positive Mental Health [50].

### 2.5. Intervention Protocol

Allocation of participants in the program: Nurses recruited participants in the chronic patient consultations of the Primary Care Centre through consecutive case sampling as they came to the nurses’ office. Once included in the study, participants were scheduled for a first visit where, after signing the informed consent, sociodemographic variables were collected using the ad hoc form, and variables of adherence to the Mediterranean diet, level of sedentary/physical activity, and level of Positive Mental Health were collected according to the validated instruments. Participants enrolled in the study were blindly randomized into two groups of 30 people each. Randomization was performed using a database that assigned phase I participants a number, which could be 1 or 2, alternately stratified by sex. The participants in the intervention group were contacted by telephone and were scheduled at the hospital center where the specialists were located.

Program procedures: The intervention group participants underwent a pre-test assessment. This initial evaluation was carried out at the Sports Medicine and Physical Activity unit on two separate days. On the first day, the physical activity specialist nurse performed the blood tests and advanced anthropometry. On the second day, the stress test was conducted by a sports physician and a cardiologist. The following week, the participants were scheduled to meet with the nutritionist, who assessed the body composition of the participants with the TANITA scale and collected their eating habits with an ad hoc questionnaire. Throughout the intervention, which lasted nine months, participants received follow-ups every two months by specialists, to review the goals and establish new ones. Physical activity data were recorded daily in the patient’s notebook, where the patient noted whether the goal was achieved (had met the target), partially achieved (met it on at least one day of the week), or not achieved (had not met the target). Mobility was recorded using the Google FIT mobile application. At each visit, the specialists collected the data in an Excel file, which was only accessible to the research team. At the end of the intervention, the intervention group received a post-test assessment for nutritional and body composition variables (ad hoc food consumption frequency questionnaire, bio impedance with the TANITA scale, and advanced anthropometry), physical fitness variables (stress test), and analytical parameters (blood test). The sequence for collecting these data was the same as that carried out at the beginning of the program.

The control group received an obesity follow-up visit with the nurse at the Primary Care Centre. The visits were conducted on a bi-monthly basis, during which participants were weighed and were provided with information and important guidance pertaining to physical activity and healthy nutritional habits.

Upon completion of the “Cerdanya en Forma” program, the variables for physical activity levels, adherence to the Mediterranean diet, and the level of Positive Mental Health were assessed in both the intervention and control groups to determine whether there were any posttest differences between the two groups. The questionnaires were collected by the research team and entered into a database. 

### 2.6. Statistical Analysis

In the descriptive analysis, frequencies and percentages were used for qualitative variables and the mean (standard deviation) and median (maximum–minimum interquartile range) for quantitative variables. The homogeneity of the two groups (intervention and control) was verified using Fisher’s chi-square or the exact test for qualitative variables and the Mann–Whitney U test for quantitative variables. Subsequently, the paired *t*-test was used for each of the groups separately and a random effects model (intercept) with interaction was fitted to test whether there were pre-post changes and whether these changes were different in the two groups for the variables of adherence to the Mediterranean diet and Positive Mental Health. In the pre-post analysis of the intervention group in the stress ergometry variables, the Wilcoxon test and the exact symmetry test were used. For the metabolic and body composition variables, the Wilcoxon test was also used, except for weight and BMI, which were tested with a mixed-effects linear regression model. A *p* value of < 0.05 was considered statistically significant. IBM SPSS Statistics, version 17.0 was used for the statistical analysis.

## 3. Results

**Figure 1:**
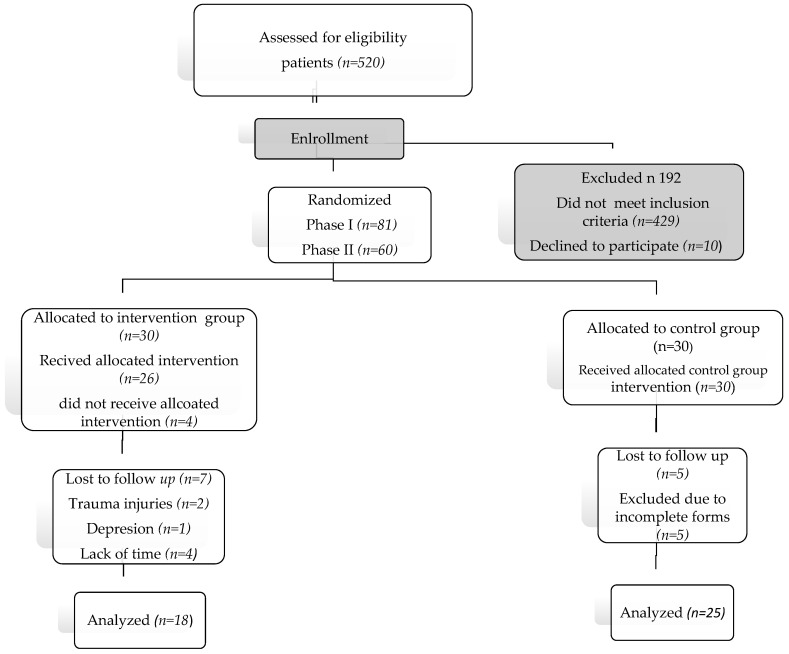
Flow diagram of study participants.

Of 520 individuals who were assigned to the Primary Care Centre, 429 failed to meet the inclusion/exclusion criteria. Of the 91 eligible participants, 10 declined to participate in the study. In the first phase, 81 subjects were analyzed. In phase II, 60 subjects from phase 1 were randomized into 2 groups, including 30 subjects in the intervention group and 30 subjects in the control group. See Figure 1.

### 3.1. Description of the Sample (Baseline)

Total sample:

The mean age of the sample was 52 years (SD: ±13.5), 71.4% (n = 57) of which were women. Approximately 49.4% (n = 40) had primary education, 37.7% (n = 30) had secondary education, and 13% (n = 10) had higher education. In total, 63.3% (n = 51) of participants were married, 19.5% (n = 16n) were single, 13% (n = 10n) were divorced, and 5.2% (n = 4) were widowed. Regarding employment status, 51.9% (n = 42) of participants had a permanent contract, 3.9% (n = 3) were temporarily employed, 27.3% (n = 22) were unemployed, and 16.9% (n = 14) were retired. In terms of country of origin, 66.2% (n = 54) of participants were Spanish, 31.8% (n = 26) were South American, and 1.3% (n = 1) were from Morocco.

Regarding health variables, the mean weight of participants in kg was 92.3 kg (SD: ±12.8), with a mean height of 162 cm (SD: ±23.4) and a mean BMI of 34.4 (SD: ±3.1). Approximately 36.4% (n = 30) developed obesity in adulthood, 32.5% (n = 26) did so during maternity, and 29.9% (n = 24) became obese during childhood and/or adolescence. Concerning cardiovascular risk factors, the prevalence of hypertension was 40.3% (n = 33), whereas the incidence of diabetes was 23.7% (n = 19). Dyslipidemia was observed in 42.9% (n = 34) of the population, and 7.8% (n = 6) of the cohort had been diagnosed with ischemic heart disease. Furthermore, 58.7% (n = 47) of the sample had additional medical conditions, and 66.2% (n = 54) were taking medication, of which 12% (n = 10) were beta-blockers. In total, 16.9% (n = 14) of the sample identified themselves as current smokers.

Randomized sample: Intervention and control groups:

The baseline sociodemographic and health characteristics of the randomized sample participants in the control group (n = 25) and intervention group (n = 18) revealed no significant differences. The mean age was 49.6 years (SD: ±12.5); the majority were women, and half of the participants had primary education. Half of the participants had secure employment, and most were of Spanish nationality. Regarding health variables, the mean weight of both groups was 93.3 kg (SD: ±14.2), with a BMI of 34.6 (SD: ±3.1).

Most of the participants developed obesity during maternity and/or adulthood. One third of both groups had hypertension and dyslipidemia; half had other chronic diseases and were taking medication regularly. The majority was non-smokers and were not taking beta-blockers. No statistically significant differences were observed across the three dependent variables studied in the baseline data. Regarding the assessment of adherence to the Mediterranean diet, both groups obtained a mean score of 8 (low adherence) (*p*-value = 0.34). The assessment of the level of physical activity at work and in leisure time was similar in both control and intervention groups: sedentary patients 8% (n = 2) vs. 10.5% (n = 2), minimally active patients 32% (n = 8) vs. 42% (n = 8), slightly active patients 36% (n = 9) vs. 36.1% (n = 7), and moderately active patients 20% (n = 5) vs. 10% (n = 2) (*p*-value = 0.85). On the Positive Mental Health Scale, the mean score was 120 (SD: ±17.7) (*p*-value = 0.66). Upon analyzing the different factors of the scale, there were no significant differences between control and intervention groups in the pretest. F1 (*p*-value = 0.41), F2 (*p*-value = 0.38), F3 (*p* value = 0.25), F4 (*p*-value = 0.20), F5 (*p*-value = 0.53), and F6 (*p*-value = 0.39).

### 3.2. Multidisciplinary Intervention Program: “Cerdanya en Forma”

Changes in the frequency of physical activity and physical condition:

The pre-post-test results when comparing the intervention and control groups regarding the increase in the frequency of physical activity were significant in favor of the intervention group (*p* value < 0.001 vs. 0.865). Thus, the intervention group participants went from being 89.4% (n = 16) not very active/sedentary in the pretest to 94.4% (n = 17) active in the posttest. At the posttest, there were no poorly-active/sedentary participants. The changes in physical condition in the pre-post test of the intervention group assessed with stress ergometry are shown in Table 1. No significant changes in terms of aerobic threshold or VO_2_ Max were identified, although there were pre-post test changes in the improvement of physical condition.

Adherence to the Mediterranean diet after the intervention:

In terms of the observed pre-post test changes concerning adherence to the Mediterranean diet between groups, the intervention group increased 2.2 points on average on the scale [mean: 10.5 (CI 95%: 9.90; 11.09)], obtaining good adherence to the Mediterranean diet in the post-test median of 8 points (IQR 7–10) vs. the median of 10 points (IQR 10–11). In the control group, no changes were observed in the scale scores [median 7 (IR 6–9) vs. median 7 (IQR 7–9)]. Paired *t*-test and linear regression statistical tests indicate that there are significant differences between intervention and control groups (*p* value = 0.00 vs. 0.81).

Positive Mental Health (PMH) after the intervention:

In the PMH scale, no significant differences were observed between the control and intervention groups for the following factors: factor 2 (pro-social attitude), factor 3 (self-control), factor 4 (self-esteem), and factor 6 (interpersonal relationship skills). However, significant differences were observed in both groups for factor 1 (personal satisfaction), factor 5 (problem solving and updating), and the overall score of the PMH scale. The pre-post test results of both groups are shown in Table 2.

### 3.3. Metabolic and Anthropometric Changes after the Intervention

The anthropometric parameters of the intervention group improved significantly. A reduction in the percentage and kg of body fat [mean: −2.50 kg (CI 95%: −3.56, −1.44)], free fat mass [mean: −3.38% (IC 95%: −4.34, −2.41), and the sum of skin-folds [mean: −25.53 cm (CI 95%: −32.80, −18.279] was observed. In addition to a decrease in cm of waist–hip circumference, the kg of muscle and bone mass increased. Weight [mean: −5.92 kg (CI 95%: −8.53, −3.32)] and BMI [mean: −2.24 kg/m^2^ (CI 95%: −3.16, −1.31)] also reduced significantly in all the tests performed during the program. As for the analytical parameters, the blood glucose profile was not significant since both the pre- and post-test blood glucose values of the participants were within normal ranges. In contrast, significant changes were observed in the decrease in triglyceride [mean: −20.87 mg/dL (CI 95%: −37.49, −4.26)] and total cholesterol levels [mean: −11.52 mg/dL (CI 95%: −21.30, −1.74) and in the increase in High-Density Lipoprotein (HDL), representing an increase in metabolic expenditure. Although Low-Density Lipoprotein (LDL) values were lower in the posttest, no significant pre-post test differences were observed. The pre-post test results for the metabolic and anthropometric variables are shown in the following table. See Table 3.

### 3.4. Participant Satisfaction with the Program

One hundred percent of the participants who completed the program (n = 18) stated they would repeat the experience and recommend it. Furthermore, 90% (n = 16) of participants reported feeling very satisfied with the program, whereas 10% (n = 2) were quite satisfied. When asked about the perceived improvement in physical condition, 100% said that they had improved their physical condition. Regarding eating habits, 94.4% (n = 17) considered that they ate healthier. In terms of the acquisition of nutritional habits and Positive Mental Health techniques, 83.4% (n = 15) responded that they had learned quite a lot, whereas 16.7% (n = 3) responded not very much. Despite these findings related to learning, only 66.7% (n = 12) felt that they would be able to autonomously perform the techniques learned. Finally, 100% (n = 18) of the participants considered that the program had been useful for improving their health.

## 4. Discussion

In this study, the mean age of participants was 52 years (SD: ±13.5), and 58.7% (n = 47.6) of the total sample were people with multiple pathologies, with 62.2% (n = 50) on medication. In addition to having cardiovascular risk factors, such as high blood pressure (40.3%; n = 33) and dyslipidemia (42.9%; n = 34), 23.7% (n = 19) had diabetes and 7.8% (n = 6) had ischemic heart disease. Considering that 82% (n = 66.4) were sedentary or minimally active (<600 METS/min/week), there is a high risk of premature aging and comorbidity [54,55,56]. Moreover, according to Pang Wen et al. (2011), inactive or sedentary people have a 17% higher mortality risk than active people [57].

### 4.1. Multidisciplinary Intervention: “Cerdanya en Forma”

Changes in the frequency of physical activity and physical condition:

The scientific literature has shown that the prescription of physical activity including frequencies, min, METs, or steps in sedentary and inactive patients is effective for improving physical activity levels and contributing to greater physical, emotional, and social well-being [58,59]. The physical activity strategy of the intervention in our study showed that 88.8% (n = 16) of the individuals with obesity evolved from being sedentary or not very active to being active or very active at the end of the program. The level of physical activity was measured using a pedometer [60,61], and active breaks were provided with the objective of disrupting the sedentary lifestyle. The physical activity strategy was effective at increasing the level of physical activity in individuals with obesity who underwent the intervention [62]. In addition, the effort test showed an improvement in physical fitness, as evidenced by an increase in H R max and endurance to overcome the stages of the Bruce cardiovascular test where the intensity (km x hour and percentage of inclination) was increased every 3 min. However, the results do not show a significant improvement in the aerobic threshold, although recent studies affirm that improved cardiovascular resistance minimizes the risks of non-communicable diseases such as obesity or ischemic heart disease [5,63,64].

The program presented in this study also includes a nutritional strategy to enhance dietary habits and a Positive Mental Health strategy to facilitate psychoemotional management. A meta-analysis showed that addressing obesity with multimodal interventions improves dietary intake and physical activity levels [65]. Differences between groups for dietary outcomes were of the magnitude of 65 kcal/day (control group) vs. −500 kcal/day in the intervention group [59]. Thus, the changes observed in relation to the improvement of health status are a consequence of the three interventions, in line with other studies that perform both dietary, physical activity, and behavioral interventions [44,66,67].

Adherence to the Mediterranean diet after the intervention:

In relation to the participants’ adherence to the Mediterranean diet, the results indicate that the intervention group went from having a low adherence in the pretest to having a good adherence in the posttest, supporting an improvement in the eating habits of the intervention group participants. These results are in line with other studies where nutritional interventions were carried out [68]. Senger et al. (2017), in their systematic review, point out that behavioral interventions in nutritional habits at 6–12 months of the evolution improve eating habits [4]. In addition, in the program satisfaction questionnaire regarding eating habits, 94.4% (n = 17) of the intervention group participants said they ate healthier at the end of the program, and 83.4% (n = 15) said they had learned to eat healthily, although only 66.7% (n = 12) stated that they were able to continue healthy eating habits autonomously, without nutritional support.

Positive Mental Health (PMH) after the intervention:

At the psychoemotional level, the intervention group participants showed a tendency to improve PMH, although no significant pre-post test differences were observed. This may be because participants in the intervention group exhibited a favorable mental health profile at the outset of the study. Also, the psychoemotional intervention was based on four sessions during the entire program. However, not all participants attended all these sessions. Consequently, it would be beneficial to conduct additional sessions to evaluate significant changes at the emotional level. Different studies point out that emotional interventions improve adherence to acquired nutritional and physical activity habits, enhance emotional management, and maintain the decrease in BMI for a longer period in patients with obesity [68,69]. Moreover, if good mental and emotional health is not maintained, it may be difficult to sustain the lifestyle changes acquired in the program. Participants should also be emotionally monitored and supported through the process of change [70].

### 4.2. Metabolic Cardiovascular Risks and Changes in Body Composition

Comparing the results of the study with the literature reviewed in relation to the glucose profile, no statistically significant changes can be observed in the pre-post test since most of the study participants had glycaemia within normal parameters at baseline, which does not mean that physical activity did not decrease insulin resistance as suggested by the literature [30]. Concerning the lipid profile, a decrease was observed in the three parameters under study: total cholesterol [mean: −11.52 mg/dL (CI 95%:−21.30, −1.74), triglyceride [mean: −20.87 mg/dL (CI 95%: −37.49, −4.26)], and LDL [mean: −7.27 (CI 95%: −15.53, 0.98)]; while mean LDL levels declined, the change was not significant—this may also be attributable to the small sample size or large variability in LDL levels. Furthermore, in addition, an increase in HDL indicates that energy demand has increased in line with the increase in physical activity. Viadas et al. (2023) evaluated the dose–response relationship between physical activity and HDL functionality parameters. The results indicate a non-linear association with increased HDL antioxidant capacity [71]. The values of these metabolic parameters can be related to the findings observed in body composition [44]. Similar to other studies examining the relationship between physical activity and nutritional interventions with metabolic changes such as: insulin resistance index or cholesterol; alterations in cardiovascular risk factors, such as hypertension; and BMI or weight reduction [44,45], participants in the intervention group demonstrated a reduction in percentage and kg of body fat [mean: −2.50 kg (CI 95%: −3.56, −1.44)] and percentage of fat-free mass [mean: −3.38% (IC 95%:−4.34, −2.41), accompanied by a marked increase in kg of muscle [mean: 1.09 (CI 95%: 0.41, 1.78)] in the posttest. Furthermore, there was a reduction in abdominal circumference [mean: −4.25 cm (CI 95%: −5.84, −2.66)], accompanied by a decrease in waist-to-hip ratio, which is an index of cardiovascular risk [4]. Changes in body composition parameters are also reflected in the literature in interventions involving continuous physical activity, based on intermittent physical exercise combined with healthy and non-restrictive dietary measures [45].

## 5. Limitations

The current study presents several limitations worth considering. First, the absence of metabolic parameters in the control group (including glucose, cholesterol levels, cardiovascular risk, and oxygen consumption) precludes the possibility of making comparisons with the intervention group. This was because not all participants in the control group consented to the collection of these biological parameters prior to receiving the intervention. A second limitation is the small sample size due to the population density of the specific area where the study was conducted since it is a rural area. Finally, this study did not assess the maintenance or adherence to the lifestyle change in the long term. This study should be replicated in larger and more diverse populations, and the impact of the intervention should be evaluated in the long term. Moreover, the impact of interdisciplinary interventions in various groups at risk of obesity has not yet been evaluated. Therefore, it would be advisable to conduct an in-depth study of the groups that are most susceptible to develop obesity to corroborate the results and support the need for such interventions.

## 6. Clinical Practice

This study provides a comprehensive vision of the person by treating the different dimensions that affect chronic pathology as a whole, specifically, in this case, obesity. A multimodal and/or interdisciplinary intervention model was used that is transferable and applicable to the current health system. Professionals must work cross-cuttingly to empower people with obesity to take care of their health.

## 7. Conclusions

The “Cerdanya en forma” program improves the overall lifestyle of people with mild to moderate obesity, promoting an active lifestyle, healthy eating, and Positive Mental Health. The people who participated in the program increased their level of activity and physical condition, resulting in an increase of muscle mass, as well as a reduction in the kg of fat, especially at the abdominal level, consequently reducing cardiovascular and metabolic risk factors. They also increased their adherence to the Mediterranean diet and their emotional well-being. Thus, the combination of the four parameters (PA, Diet, PMH, and metabolic changes) is an innovative and complementary alternative that provides care from a holistic perspective. Nevertheless, further research is necessary with diverse risk groups related to obesity to confirm these findings.

## Figures and Tables

**Table 1:** Pre-post Bruce Treadmill fitness test differences of the intervention group.

Variables	Pretest (n = 18) Mean (IC 95%) Median IQR [25, 75]	Posttest (n = 18) Mean (IC 95%) Median IQR [25, 75]	Pre-Post Test Difference Mean (IC 95%) Median IQR [25, 75]	*p*-Value
**Bruce stage**	2.11(1.77, 2.45) 2.00 [2.00, 3.00]	2.56 (2.21, 2.91) 2.00 [2.00, 3.00]	0.44 (0.19, 0.70) 0.00 [0.00, 1.00]	0.005
**Seconds in the stage**	75.56 (45.27, 105.84) 50.00 [30.00, 120.00]	66.94 [(41.46, 92.43) 55.00 [30.00, 110.00]	−8.61 (−41.66, 24.44) 0.00 [−60.00, 30.00]	0.844
**Max HR (ppm)**	155 (144.96, 165.05) 154 [154.00, 172.00]	160.33 (150.27, 170.39) 163.50 [144.00, 178.00]	5.33 (−0.64, 11.30) 6.50 [−1.00, 12.00]	0.05
**Aerobic level (ppm)**	111.50 (104.60, 118.40) 110.00 [105.00, 118.00]	114.50 (107.70, 121.30) 118.50 [104.00, 122.00]	3.00 (−1.64, 7.64) 5.00 [−3.00, 11.00]	0.183
**Ve Max (L/m)**	73.32 (60.94, 85.69) 64.80 [54.70, 91.00]	78.20 (64.16, 92.24) 69.50 [52.40, 101.00]	4.88 (−1.01, 10.78) 3.85 [−3.10, 10.70]	0.112
**VO_2_ Max (L/m)**	2.00 (1.69, 2.32) 1.77 [1.50, 2.42]	2.11 (1.77, 2.44) 1.96 [1.64, 2.63]	0.10 (−0.02, 0.23) 0.13 [−0.02, 0.26]	0.093

Note. Data are mean (confidence interval 95%) and median and interquartile range M [IQR P25, P75]; *p*-value was derived from Wilcoxon’s rank sum test; Ve Max: maximum expiratory flow; VO_2_ Max: maximum oxygen consumption; Max HR: maximum heart rate; and ppm: pulses per minute. Wilcoxon signed-rank Test.

**Table 2:** Positive mental health changes in the intervention group after applying the program.

Mean (IC 95%) Median IQR [25, 75] Variables	Intervention Group (n = 18)		Control Group (n = 25)		IG	CG	IG	CG
	Pre-T	Post-T	Pre-T	Post-T	Pre-Post T Difference	Pre-Post T Difference	*p*-Value	
**F1 (PMH)** **self-esteem**	23 (20.54, 25.45) 23.5 [20, 27]	25.44 (23.22, 27.66) 25.5 [23, 30]	23.8 (21.4, 26.25) 25 [21, 28]	25.82 (24.34, 27.30) 26 [24, 28]	−2.44 (−4.28, −0.60) −3 [−4, −1]	−2 (−4.01, 0.015) −1 [−3; 1]	0.008	0.048
**F2 (PMH)** **self-control**	17.77 (16.69, 18.86) 18.5 [17, 19]	17.61 (16.58, 18.63) 19 [16, 19]	6.8 (15.59, 18.14) 17 [16, 19]	7.34 (16.36, 18.32) 18 [16, 19]	0.16 (−1.054, 1.38) 0 [−2, 2]	−0.478 (1.43, 0.478) 0 [−2, 1]	0.930	0.346
**F3 (PMH)** **proactive attitude**	15.27 (13.41, 17.14) 16 [13, 19]	15.22 (13.71, 16.73) 16 [13, 17]	13.82 (12.27, 15.37) 14 [11, 17]	14.04 (12.78, 15.30) 14 [12, 16]	0.055 (−1.35, 1.46) 0 [−2, 1]	−0.217 (−1.60, 1.17) 0 [−1, 2]	0.642	0.975
**F4 (PMH)** **proactive attitude**	16.16 (14.11; 18.22) 17.5 [14, 19]	16.72 (15.26, 18.17) 17.5 [16, 19]	15.21 (14.05, 16.38) 16 [14, 17]	15.69 (14.64, 16.74) 17 [14, 17]	−0.55 (−1.81, 0.70) 0 [−3, 0]	−0.478 (−1.59, 0.64) 0 [−1, 0]	0.527	0.538
**F5 (PMH)** **personal satisfaction**	27.22 (24.73, 29.7) 28 [25, 31]	31.5 (29.80, 33.19) 32 [31, 34]	26.56 (24.47, 28.65) 27 [25, 30]	29.26 (27.25, 31.26) 31 [27, 33]	−4.27 (−5.51, −3.03) −4.5 [−6, −2]	−2.69 (−4.77, −0.61) −2 [−4, −1]	0.000	0.006
**F6 (PMH)** **interpersonal relationship skills**	22.94 (21.37, 24.51) 23 [21, 25]	24 (22.73, 25.26) 24.5 [22, 26]	21.39 (19.56, 23.21) 23 [17, 25]	22.17 (20.58, 23.75) 23 [21, 24]	−1.05 (−2.58, 0.47) −1 [−3, 1]	−0.782 (−2.32, 0.75) 0 [−2, 2]	0.100	0.461
**Total PMH**	122.38 (114.34, 130.43) 123 [113, 133]	130.5 (124.39, 136.60) 133 [125, 138]	117.69 (109.19, 126.19) 124 [111, 132]	124.34 (117.33, 131.36) 129 [121, 135]	−8.11 (12.67, −3.54) −7.5 [−13,−3]	−6.65 (−13.70, 0.39) −3 [−11; 1]	0.003	0.041

Note. Data are mean (confidence interval 95%) and median and interquartile range M [IQR P75, P25]; *p*-value was derived from Wilcoxon Signed rank test. PMH: Positive Mental Health.

**Table 3:** Metabolic and anthropometric changes after the intervention.

Mean (IC 95%) Median [p25, 75]	Pretest (n = 18)	Posttest (n = 18)	Pre-Post Difference	*p*-Value
**Glucose Profile (mg/dL)**	102.08 (97.49, 106.67) 103.00 [95.00, 106.60]	99.83 (94.78,104.88) 98.90 [91.70,103.00]	−2.25 (−6.45, 1.95) −3.00 [−8.00, 1.70]	0.112
**Total cholesterol (mg/dL)**	194.28 (180.16, 208.40) 196.00 [172.60, 211.70]	182.77 (167.77, 197.76) 191.25 [154.00, 205.00]	−11.52 (−21.30, −1.74) −8.75 [−23.00, 1.00]	0.028
**HDL(mg/dL)**	53.59 (45.15, 62.04) 49.70 [45.90, 54.10]	60.79 (51.60, 69.99) 52.90 [49.90, 75.60]	7.20 (1.63, 12.77) 4.55 [1.00, 9.60]	0.003
**LDL (mg/dL)**	121.60 (109.24, 133.96) 120.80 [104.00, 140.30]	114.33 (106.70, 121.96) 116.70 [101.60, 124.30]	−7.27 (−15.53, 0.98) −7.15 [−19.00, 4.40]	0.157
**Triglycerides (mg/dL)**	142.04 (104.92, 179.15) 116.25 [87.00, 185.50]	121.17 (88.77, 153.56) 106.35 [81.70, 138.00]	−20.87 (−37.49, −4.26) −8.90 [−39.10, 3.30]	0.033
**% body fat**	43.63 (41.16, 46.10) 45.40 [37.90, 46.80]	42.68 (40.20, 45.16) 44.60 [36.40, 45.70]	−0.95 (−1.30, −0.60) −0.85 [−1.20, −0.40]	0.000
**Kg body fat**	39.98 (37.19,42.78) 39.95 [36.60, 43.60]	37.48 (34.68, 40.29)] 37.80 [35.50, 39.80]	−2.50 (−3.56, −1.44) −1.80 [−2.80, −1.30]	0.000
**Free fat mass**	41.76 (38.22, 45.29) 45.20 [36.70, 46.60]	38.38 (34.72, 42.05) 41.20 [31.50, 44.20]	−3.38 (−4.34, −2.41) −3.50 [−5.20, −1.50]	0.000
**Sum of folds (mm)**	112.06 (99.14, 124.98) 115.00 [91.00, 128.00]	86.52 (72.84, 100.20) 80.15 [68.00, 107.60]	−25.53 (−32.80, −18.27) −27.50 [−33.00, −16.00]	0.000
**% of water**	41.81 (39.33, 44.28) 38.90 [38.60, 46.50]	43.72 (41.33, 46.12) 42.10 [39.80, 48.90]	1.92 (1.38, 2.45) 2.10 [1.00, 2.50]	0.000
**% muscle**	27.76 (26.34, 29.17) 27.65 [26.20, 29.40]	30.03 (28.69, 31.38) 30.15 [27.70, 32.00]	2.28 (1.46, 3.10) 2.35 [1.00, 3.30]	0.000
**Kg of muscle**	25.86 (22.94, 28.78) 25.15 [21.60, 31.10]	26.96 (23.84, 30.07) 26.50 [22.40, 32.50]	1.09 (0.41, 1.78) 1.05 [−0.10, 2.30]	0.010
**BMI (kg/m^2^)**	35.20 (33.59, 36.81) 35.50 [33.00, 36.00]	32.96 (31.33, 34.60) 32.20 [29.90, 34.70]	−2.24 (−3.16, −1.31) −1.90 [−3.30, −1.20]	0.000
**Weight (kg)**	95.13 (86.66, 103.60) 97.30 [85.80, 107.30]	89.21 (80.96, 97.46) 89.40 [81.50,101.80]	−5.92 (−8.53, −3.32) −4.00 [−10.50, −1.80]	0.000
**Waist circumference (cm)**	103.91 (98.65, 109.16) 104.50 [94.00, 108.80]	99.66 (94.67, 104.64) 99.25 [91.80, 107.00]	−4.25 (−5.84, −2.66) −3.50 [−4.20, −2.00]	0.000
**Hip circumference (cm)**	116.59 (111.91, 121.28) 116.75 [110.50, 121.50]	113.65 (109.26,118.04) 112.75 [110.00, 119.30]	−2.94 (−4.94, −0.95) −1.60 [−5.00, 0.00]	0.005
**Waist/hip ratio**	0.89 (0.84, 0.95) 0.88 [0.81, 0.99]	0.88 (0.83, 0.93) 0.86 [0.79, 0.97]	−0.01 (−0.02, −0.01) −0.01 [−0.03, 0.00]	0.004

Note. Data are mean (confidence interval 95%) and median and interquartile range M [IQR P75, P25]; *p*-value was derived from Wilcoxon Signed rank test. HDLs: High-Density Lipoproteins; LDLs: Low-Density Lipoproteins.

## Data Availability

The data that support the findings of this study are available from the corresponding author upon reasonable request.

## Abbreviations

WHO	World Health Organization
BMI	Body Mass Index
PMH	Positive Mental Health
NCD	Non-communicable disease
HDL	High-Density Lipoprotein
LDL	Low-Density Lipoprotein
SD	Standard deviation
IQR	Interquartile range
